# A prognostic nomogram based on risk assessment for invasive micropapillary carcinoma of the breast after surgery

**DOI:** 10.1002/cam4.5595

**Published:** 2023-01-05

**Authors:** Yuyuan Chen, Caixian Yu, Dedian Chen, Yiyin Tang, Keying Zhu, Rong Guo, Sheng Huang, Zheng Li, Lvjun Cen

**Affiliations:** ^1^ The Second Department of Breast Surgery The Third Affiliated Hospital of Kunming Medical University Kunming China; ^2^ The Department of Thyroid and Breast Surgery The Affiliated Hospital of Medical School of Ningbo University Ningbo China; ^3^ The Department of Gynecology Oncology The Third Affiliated Hospital of Kunming Medical University Kunming China

**Keywords:** breast cancer, invasive micropapillary carcinoma, nomogram, overall survival, surgery

## Abstract

**Purpose:**

Invasive micropapillary carcinoma (IMPC) is one of the rare subtypes of breast cancer. This study aimed to explore a predictive nomogram model for IMPC prognosis.

**Methods:**

A total of 1855 IMPC patients diagnosed after surgery between 2004 and 2014 were identified from the Surveillance, Epidemiology and End Results (SEER) database to build and validate nomogram. A nomogram was created based on univariate and multivariate Cox proportional hazards regression analysis. Receiver operating characteristic (ROC) curves were used to demonstrate the accuracy of the prognostic model. Decision curve analysis (DCA) was performed to evaluate the safety of the model in the range of clinical applications, while calibration curves were used to validate the prediction consistency.

**Results:**

Cox regression analysis indicated that age ≥62 at diagnosis, negative ER status, and tumor stage were considered adverse independent factors for overall survival (OS), while patients who were married, white or of other races, received chemotherapy or radiotherapy, had a better postoperative prognosis. The nomogram accurately predicted OS with high internal and external validation consistency index (C index) (0.756 and 0.742, respectively). The areas under the ROC curve (AUCs) of the training group were 0.787, 0.774 and 0.764 for 3, 5 and 10 years, respectively, while those of the validation group were 0.756, 0.766 and 0.762, respectively. The results of both DCA and calibration curves demonstrated the good performance of the model.

**Conclusions:**

A nomogram for IMPC of the breast patients after surgery was developed to estimate 3, 5 and 10 years—OS based on independent risk factors. This model has good accuracy and consistency in predicting prognosis and has clinical application value.

## Graphical abstract

A nomogram predicting 3, 5 and 10 year overall survival for patients with invasive micropapillary carcinoma of breast.

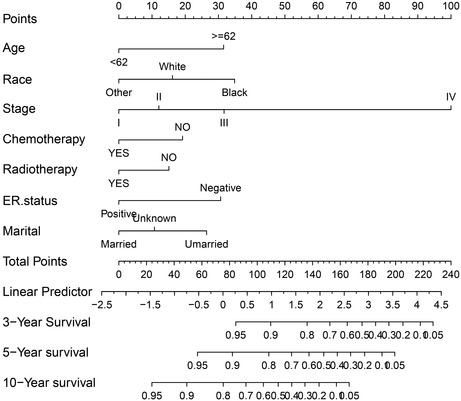

## 1 INTRODUCTION

Breast cancer (BC) is the most common malignant tumor worldwide, which seriously endangers women's lives and health.^1^ Invasive micropapillary carcinoma (IMPC) is a rare type of invasive carcinoma of the breast, accounting for only 0.9%–2% of cases.^2^, ^3^ IMPC is defined in the WHO classification of breast tumors as an invasive carcinoma with small clusters of tumor cells arranged in a mesenchymal lumen resembling a vasculature. Consisting of clusters of mulberry‐like or glandular ductal or alveolar‐like carcinoma cells, IMPC has a polarity flip phenomenon.^2^, ^4^ IMPC often coexists with invasive ductal carcinoma (IDC) and can usually be differentiated by EMA staining.^2^

The SEER (surveillance, epidemiology and end results) database is established by the National Cancer Institute to provide reliable and valuable information on cancer statistics.^5^ The nomogram prediction model provides a simple and visual representation of prognostic‐related risk factors that can guide clinical research. IMPC has biological characteristics of high lymph node metastasis, recurrence and distant metastasis.^6^ In the past, this carcinoma was considered a breast cancer with poor prognosis, but the survival rate of IMPC has increased significantly in recent years.^2^, ^7^, ^8^, ^9^ Wu et al. analyzed 881 IMPC patients from SEER and determined that IMPC had good breast cancer‐specific survival (BCSS) and OS.^10^ Recently, Meng et al. constructed a nomogram of 388 cases of IMPC and determined that age, lymph node metastasis, hormone receptor status, adjuvant radiotherapy and other factors may affect locoregional recurrence (LRR) after mastectomy.^7^

Surgery improves the prognosis and quality of life of BC patients, but there is no prognostic prediction for patients with IMPC of the breast after surgery. In this study, we aimed to develop a nomogram to identify factors associated with improved survival in patients with IMPC.

## 2 PATIENTS AND METHODS

### 2.1 Selection of patients

IMPC after surgery was identified by SEER*Stat (version 8.3.9) from 18 population‐based cancer registries. Patients were eligible for enrollment according to the following inclusion criteria: (1) histology ICD‐O‐3 (8507), (2) surgery performed, (3) patients with primary site, (4) known ER, PR status and adjusted AJCC 6th stage. The exclusion criteria were as follows: (1) detailed information lacking age, race, grade or marital status and (2) unknown T, N, M classification and breast subtype.

### 2.2 Variable declaration

Patient characteristics included basic information, histological type, grade, breast subtype, primary site, tumor size, positive regional nodes and treatments. The patient's age was cut off at 62 years, while the tumor size was reclassified as ≤20, 20–50 and >50 mm. Surgery information was categorized as breast‐conserving surgery (BCS) or mastectomy. Primary sites of tumors were divided into central portion of breast or nipple, lower‐inner/lower‐outer/upper‐inner/upper‐outer quadrant of breast and others. The subtypes of tumors were classified as HR+/HER2− (luminal A), HR+/HER2+ (luminal B), HR−/HER2+ (HER2 enriched) and HR−/HER2− (triple negative).

### 2.3 Statistical analysis

The baseline characteristics of IMPC patients after surgery were first described statistically. OS was defined as the date of diagnosis to the date of death from any cause or the date of the last follow‐up visit. The data were divided into training and validation sets in a 7:3 ratio using the “caret” package in R version 4.1.1. Survival analysis was performed using SPSS version 25.0. Kaplan–Meier survival curves were constructed for each variable with a log‐rank test. Variables with *p* < 0.05 in univariate analysis were included in Cox proportional risk regression models to identify risk factors associated with IMPC prognosis.

Nomograms were built based on multifactor analysis using the “rms” package. The performance of the nomogram was measured by the C index to judge the accuracy of the prediction results. The total score of patients in the validation set was calculated based on the corresponding column line graphs and included as a new factor in the Cox regression model. In addition, the area under the ROC curve (AUC) was also calculated to assess the performance of the prognostic model, while the “stdca” function was used in decision curve analysis (DCA) to determine the suitability of the model. Moreover, calibration curves were plotted to compare the difference between predicted survival and actual survival determined using Kaplan–Meier analysis.

## 3 RESULTS

### 3.1 Clinicopathological characteristics of the patients

A total of 1855 patients diagnosed with IMPC after surgery were included in this study. Patients were randomly divided via a 7:3 ratio into two sets: a training set (*n* = 1300) for nomogram building, and a validation set (*n* = 555) for model validation. Next, the clinical and pathological characteristics of the patients in the training set were described in detail. The median age of primary diagnosis for the entire population was 62 (22–96) years old. The majority of patients were white (78.2%), and 98.5% were female. The breast cancer subtype was HR+/HER2− (luminal A) in 52.7% of patients, HR+/HER2+ (luminal B) in 11.2%, HR−/HER2+ (HER2 enriched) in 2.8% and HR−/HER2− (triple negative) in 2.7%. Since the Seer database has only recorded HER2 status since 2010, the HER2 status was not known for patients (30.6%) before that date. A total of 41.4% of screened patients after surgery were stage I, 37.3% were stage II, 20% were stage III, and 1.3% were stage IV. A total of 52.4% of the patients received breast‐conserving surgery (BCS), and the rest underwent mastectomy. The majority of patients developed breast cancer that was located on the upper‐outer region (29.5%), and most tumors were ≤20 mm (58.4%). The proportions of patients who underwent chemotherapy and radiotherapy were 48.9% and 55.1%, respectively. The demographic and clinical characteristics of the study participants based on dataset classification are shown in Table 1.

**TABLE 1:** Characteristics of IMPC of breast patients after surgery

Variables	Total cohort		Training cohort		Validation cohort	
	*N* = 1855		*N* = 1300		*N* = 555	
	*n*	%	*n*	%	*n*	%
Age						
<62	953	51.4	655	50.4	298	53.7
≥62	902	48.6	645	49.6	257	46.3
Race						
Black	223	12	153	11.8	70	12.6
Other	189	10.2	131	10.1	58	10.5
White	1443	77.8	1016	78.2	427	76.9
Sex						
Female	1827	98.5	1281	98.5	546	98.4
Male	28	1.5	19	1.5	9	1.6
Marital status						
Unmarried	765	41.2	522	40.2	243	43.8
Married	1004	54.1	719	55.3	285	51.4
Unknown	86	4.6	59	4.5	27	4.9
Breast subtype						
HR+/HER2− (Luminal A)	960	51.8	685	52.7	275	49.5
HR+/HER2+ (Luminal B)	210	11.3	145	11.2	65	11.7
HR‐/HER2+ (HER2 enriched)	56	3	37	2.8	19	3.4
HR‐/HER2− (Triple Negative)	50	2.7	35	2.7	15	2.7
Recode not available	579	31.2	398	30.6	181	32.6
ER						
Negative	187	10.1	130	10	57	10.3
Positive	1668	89.9	1170	90	498	89.7
PR						
Negative	393	21.2	276	21.2	117	21.1
Positive	1462	78.8	1024	78.8	438	78.9
HER‐2						
Negative	1010	54.4	720	55.4	290	52.3
Positive	266	14.3	182	14	84	15.1
Recode not available	579	31.2	398	30.6	181	32.6
Stage						
I	748	40.3	538	41.4	210	37.8
II	706	38.1	485	37.3	221	39.8
III	377	20.3	260	20	117	21.1
IV	24	1.3	17	1.3	7	1.3
Laterality						
Left	941	50.7	655	50.4	286	51.5
Right	914	49.3	645	49.6	269	48.5
Surgery						
BCS	964	52	681	52.4	283	51
Mastectomy	891	48	619	47.6	272	49
Radiotherapy						
No	833	44.9	584	44.9	249	44.9
Yes	1022	55.1	716	55.1	306	55.1
Chemotherapy						
No	930	50.1	664	51.1	266	47.9
Yes	925	49.9	636	48.9	289	52.1
Tumor size						
≤20	1086	58.5	759	58.4	327	58.9
20–50	598	32.2	422	32.5	176	31.7
>50	171	9.2	119	9.2	52	9.4
Primary site						
Central portion of breast/Nipple	124	6.7	90	6.9	34	6.1
Lower‐inner	135	7.3	95	7.3	40	7.2
Lower‐outer	143	7.7	104	8	39	7
Upper‐inner	260	14	174	13.4	86	15.5
Upper‐outer	553	29.8	384	29.5	169	30.5
Other	640	34.5	453	34.8	187	33.7

### 3.2 Prognostic factors

**FIGURE 1:**
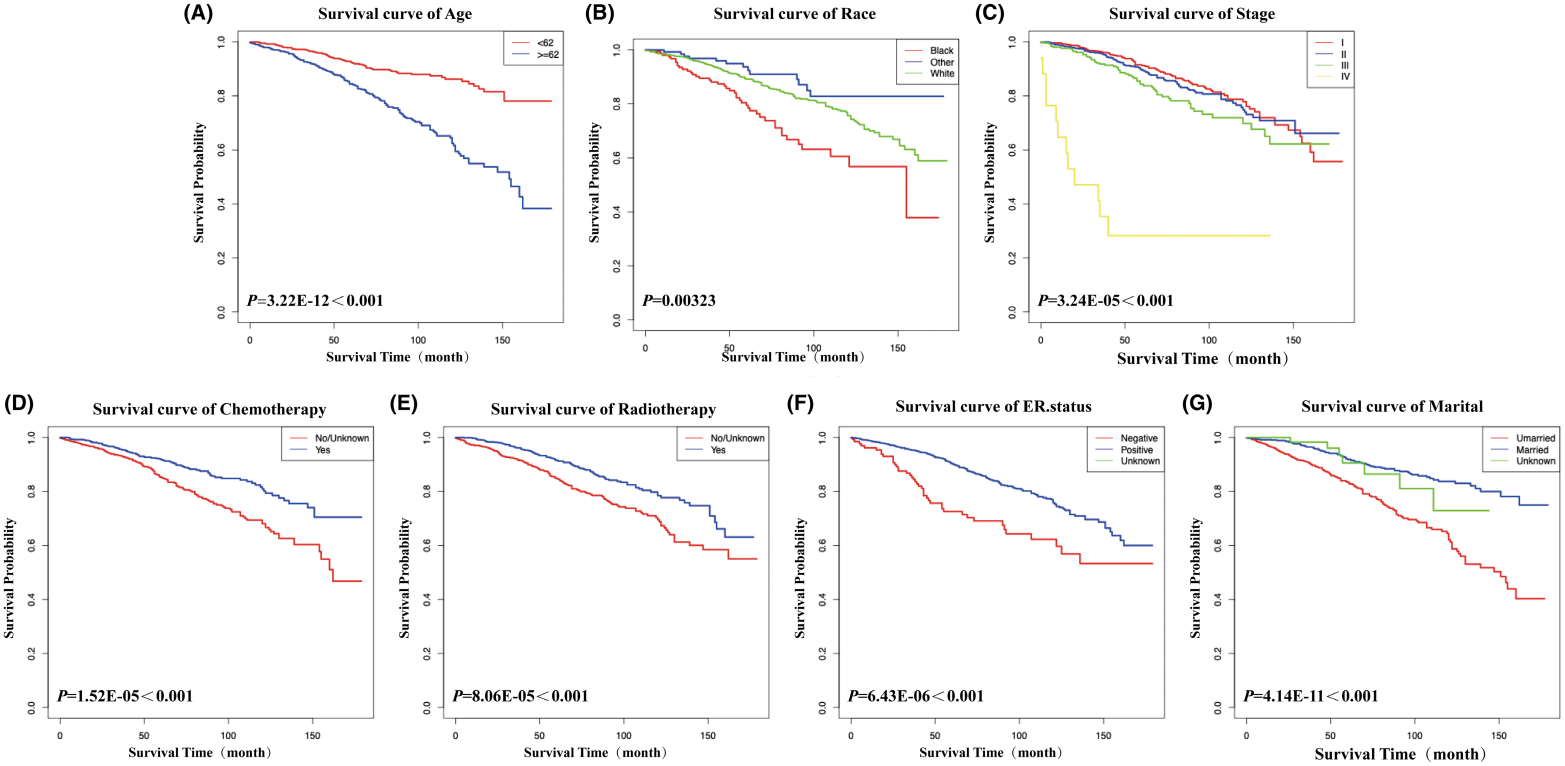
Kaplan–Meier curves of prognostic factors in patients with invasive micropapillary carcinoma (IMPC) of the breast. (A) Age at diagnosis; (B) Race; (C) AJCC 6th stage; (D) Chemotherapy; (E) Radiotherapy; (F) ER status; (G) Marital status.

As shown in Table 2, cox regression analysis was performed on the training set. Factors that were statistically significant in the univariate analysis were subjected to multiple covariance diagnosis, and strong covariance was found between T, N, M stage and clinical stage of the tumor; therefore, we did not include T, M stage and lymph node status in the cox multifactor regression model. Ultimately, age ≥ 62 (*p* < 0.001), negative ER (*p* = 0.004), stage III (*p* = 0.044), and stage IV (*p* < 0.001) were related to a significantly increased risk of IMPC patients after surgery. In contrast, marital status (*p* < 0.001), white or other race (*p* = 0.002), chemotherapy (*p* < 0.001) and radiotherapy (*p* = 0.002) were associated with a significant reduction in risk. Kaplan–Meier analysis with the log rank test was performed for the above factors using the “survival” package of R software, and the same statistical results were obtained (Figure 1). The study also found no significant difference in survival time among patients treated with two modalities of surgery. These results identified factors that may predict the occurrence of IMPC after surgery.

**TABLE 2:** Univariate and multifactorial Cox analysis of risk factors in IMPC of breast patients

Characteristics	Univariate analysis		Multivariate analysis	
	HR (95% CI)	*p* value	HR (95% CI)	*p* value
Age				
<62	Reference		Reference	
≥62	2.730 (2.057–3.622)	<0.001*	2.460 (1.812‐3.340)	<0.001*
Race				
Black	Reference		Reference	
Other	0.299 (0.161–0.556)	<0.001*	0.362 (0.192‐0.684)	0.002*
White	0.512 (0.367‐0.716)	<0.001*	0.578 (0.409‐0.816)	0.002*
Sex				
Female	Reference			
Male	1.773 (0.729–4.309)	0.206		
Marital status				
Unmarried	Reference		Reference	
Married	0.378 (0.288–0.497)	<0.001*	0.475 (0.359‐0.629)	<0.001*
Unknown	0.466 (0.218‐0.997)	0.049*	0.661 (0.307‐1.425)	0.291
Breast subtype				
HR+/HER2− (Luminal A)	Reference			
HR+/HER2+ (Luminal B)	0.833 (0.473–1.468)	0.527		
HR‐/HER2+ (HER2 enriched)	0.668 (0.211–2.113)	0.492		
HR‐/HER2− (Triple Negative)	3.891 (2.166–6.988)	<0.001*		
Recode not available	1.014 (0.737–1.396)	0.932		
Stage				
I	Reference		Reference	
II	1.082 (0.796–1.471)	0.616	1.154 (0.752–1.772)	0.511
III	1.501 (1.065–2.114)	0.020*	1.726 (1.016‐2.931)	0.044*
IV	12.909 (7.014‐23.759)	<0.001*	10.223 (4.731‐22.089)	<0.001*
T				
1	Reference			
2	1.390 (1.037–1.862)	0.027*		
3	1.674 (1.027–2.727)	0.039*		
4	6.289 (4.085–9.681)	<0.001*		
N				
0	Reference			
1	0.878 (0.642–1.201)	0.416		
2	1.003 (0.625–1.610)	0.990		
3	1.779 (1.204–2.628)	0.004*		
M				
0	Reference			
1	11.440 (6.373–20.536)	<0.001*		
ER				
Negative	Reference		Reference	
Positive	0.466 (0.335–0.650)	<0.001*	0.507 (0.322‐0.800)	0.004*
PR				
Negative	Reference		Reference	
Positive	0.587 (0.443–0.776)	<0.001*	0.792 (0.537‐1.168)	0.239
HER‐2				
Negative	Reference			
Positive	0.717 (0.428–1.201)	0.206		
Recode not available	0.912 (0.669–1.243)	0.561		
Lymph				
Negative	Reference			
Positive	1.314 (0.987–1.749)	0.062		
No examined	3.186 (2.185–4.645)	<0.001*		
Historic stage				
Localized	Reference			
Regional	0.976 (0.741–1.285)	0.864		
Distant	4.282 (2.770–6.617)	<0.001*		
Positive node				
<4	Reference			
≥4	3.098 (2.203–4.356)	<0.001*		
Unknown/no examined	1.729 (1.253–2.386)	0.001*		
Primary site				
Central portion of breast	Reference			
Lower‐inner	0.872 (0.378–2.012)	0.749		
Lower‐outer	1.683 (0.820–3.453)	0.156		
Upper‐inner	0.859 (0.418–1.764)	0.679		
Upper‐outer	1.522 (0.810–2.860)	0.191		
Other	1.169 (0.622–2.197)	0.628		
Laterality				
Left	Reference			
Right	0.883 (0.681–1.144)	0.345		
Surgery				
BCS	Reference			
Mastectomy	1.220 (0.942–1.581)	0.132		
Radiotherapy				
No	Reference		Reference	
Yes	0.592 (0.456–0.769)	<0.001*	0.652 (0.449‐0.853)	0.002*
Chemotherapy				
No/unknown	Reference		Reference	
Yes	0.554 (0.424–0.724)	<0.001*	0.557 (0.402‐0.771)	<0.001*
Tumor size				
≤20	Reference		Reference	
20–50	1.401 (1.057–1.858)	0.019*	1.339 (0.888‐2.018)	0.163
>50	2.256 (1.523–3.342)	<0.001*	1.866 (1.054‐3.303)	0.032*

* *p* < 0.05.

### 3.3 Development and validation of nomogram

**FIGURE 2:**
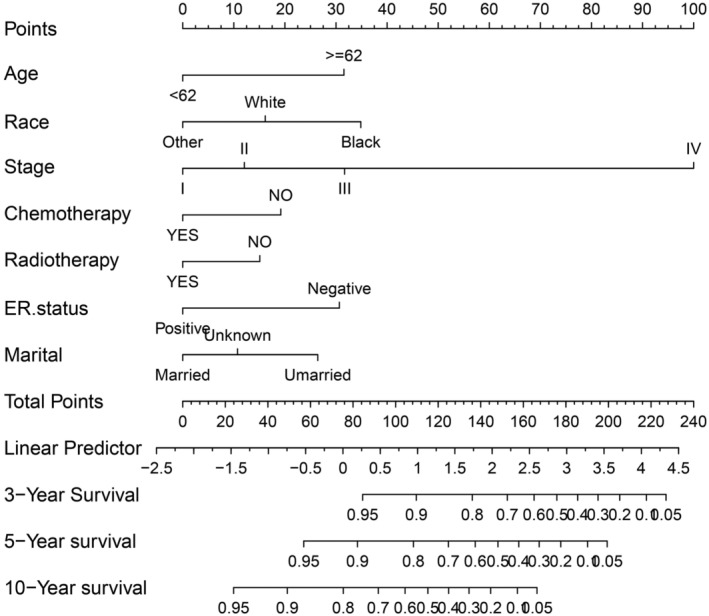
Nomogram predicting 3‐, 5‐, and 10‐year overall survival (OS) in patients with invasive micropapillary carcinoma of the breast. Rows 2–7 represent the variables incorporated into the column line graph. The scores are assigned to each variable and summed, and the total score is shown in the last row.

**FIGURE 3:**
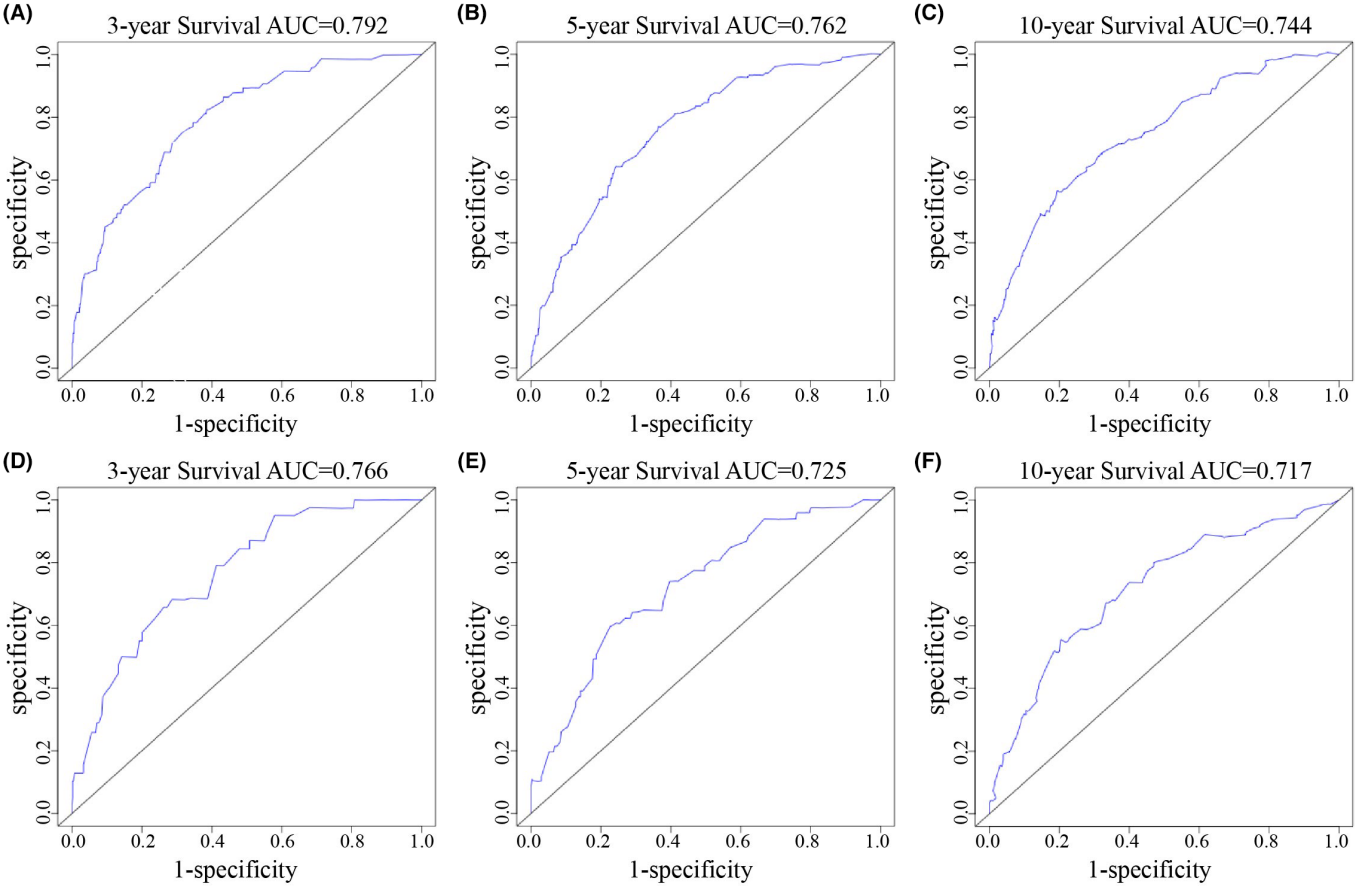
Receiver operating characteristic (ROC) curve with the area under the curve (AUC) for OS in IMPC patients. (A) 3‐year OS rate in the training set, (B) 5‐year OS rate in the training set, (C) 10‐year OS in the training set, (D) 3‐year OS rate in the validation set, (E) 5‐year OS rate in the validation set, (F) 10‐year OS rate in the validation set.

To demonstrate the interrelationship between the variables, we constructed a nomogram predicting OS by integrating independent predictors. The results of nomogram showed that stage was the main factor affecting prognosis, followed by race, age, ER status, marital status, chemotherapy, and radiotherapy (Figure 2). The prognostic models for the two groups were examined by plotting receiver operating characteristic (ROC) curves (Figure 3). The AUCs of the training group were 0.792, 0.762 and 0.744 for 3, 5 and 10 years, respectively (Figure 3A–C), while those of the validation group were 0.766, 0.725 and 0.717, respectively (Figure 3D–F). The C‐index of this nomogram was 0.756 (95% confidence interval: 0.739–0.773), and the C‐index of external validation was 0.742 (95% confidence interval: 0.717–0.767), indicating an accurate prognostic prediction of survival outcomes.

**FIGURE 4:**
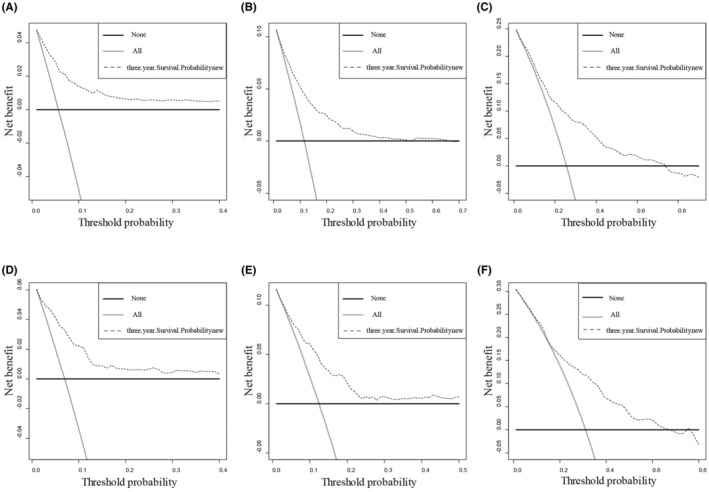
Decision curve analyses (DCA) in the training and validation sets. (A–C) Internal validation cohort, (D–F) external validation cohort. The horizontal line indicates no patient deaths, and the diagonal line indicates that all patients will have a specific death.

**FIGURE 5:**
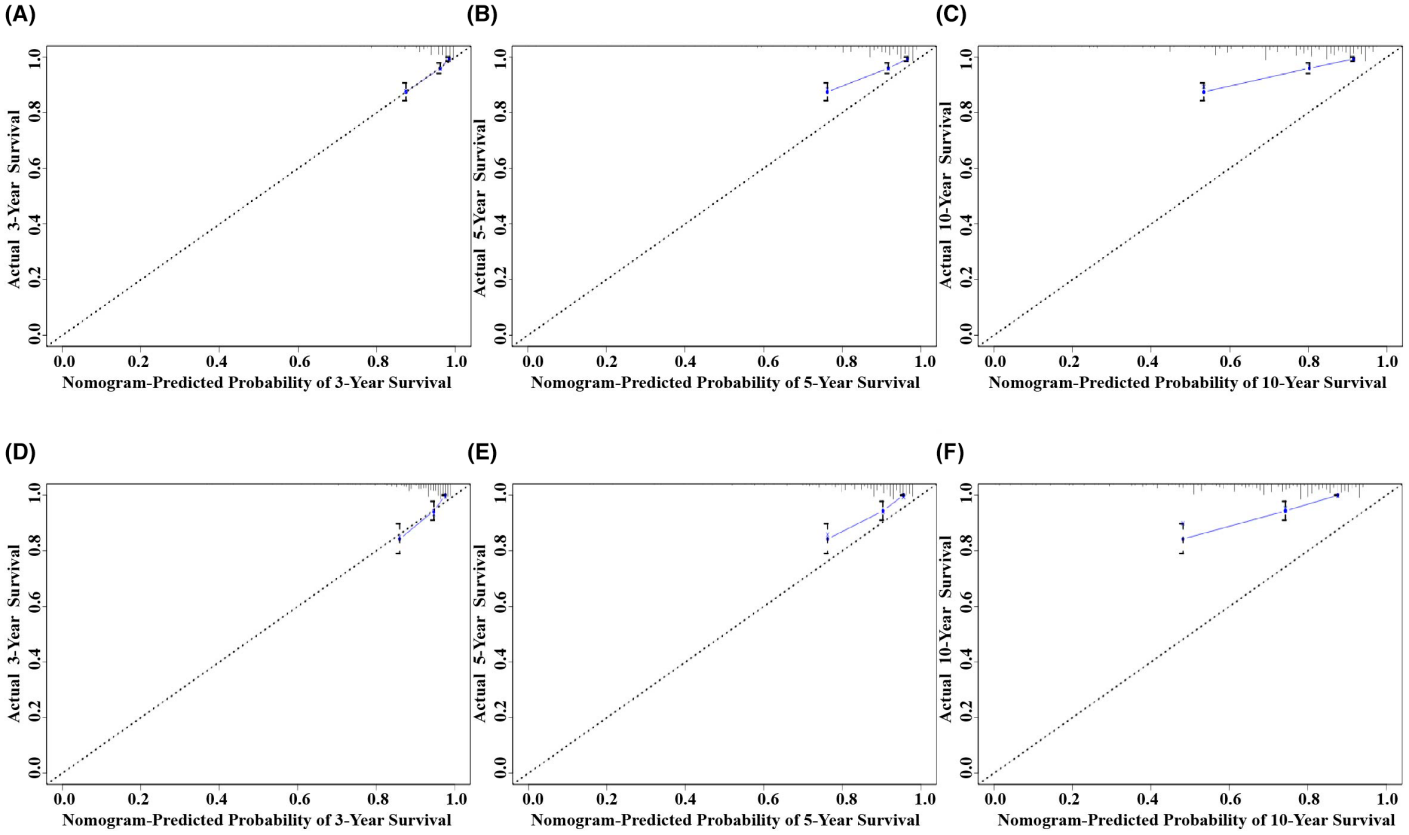
Calibration curves of the nomogram for 3‐, 5‐, and 10‐year OS prediction. (A) 3‐year OS rate in the training set, (B) 5‐year OS rate in the training set, (C) 10‐year OS in the training set, (D) 3‐year OS rate in the validation set, (E) 5‐year OS rate in the validation set, (F) 10‐year OS rate in the validation set.

Next, the calibration curves and decision curve analyses (DCA) for the 3‐, 5‐, and 10‐year OS were plotted for the training and validation sets. The generation of DCA curves validated the safety of the nomogram and has value for clinical application (Figure 4A–F). The results of the calibration curves showed a strong agreement between the predictions of the nomogram and the actual observations of the 3‐, 5‐, and 10‐year OS, indicating that the model was consistent (Figure 5A–F). In summary, this nomogram model has good clinical application value in predicting the prognosis of IMPC patients after surgery.

### 3.4 Risk assessment

**FIGURE 6:**
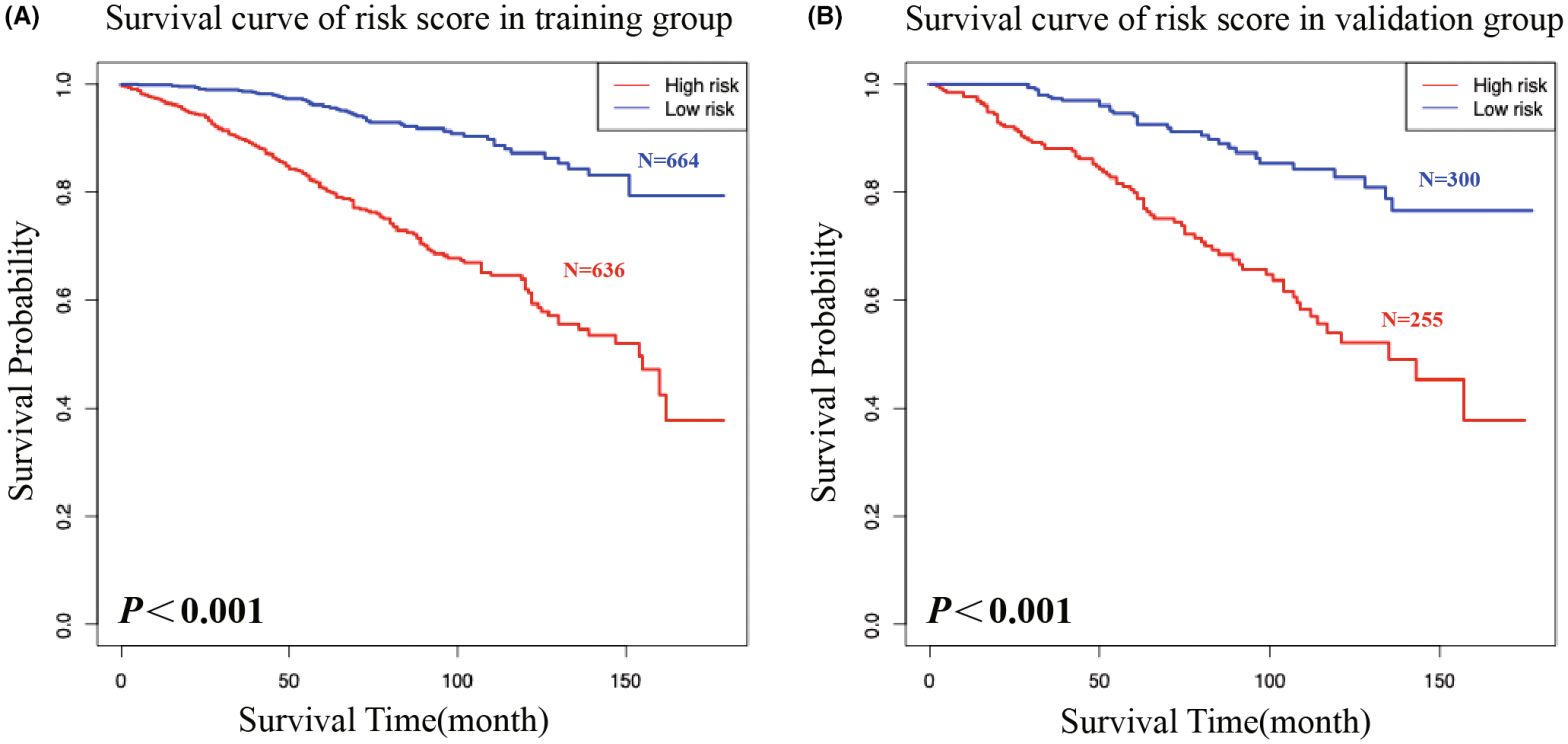
Kaplan–Meier survival analysis of patients in different risk subgroups. (A) training set, (B) validation set. The blue line represents the low‐risk group, while the red line represents the high‐risk group.

According to the OS of postoperative risk of breast cancer, we divided the patients into three groups by “coxph” function, including low‐risk group and high‐risk group. By plotting Kaplan–Meier survival curves for each group, we found that the results of both the training and validation sets showed statistically significant differences in OS for patients with different risk levels (*p* < 0.001) (Figure 6A,B). These results demonstrated the strong predictive value of this risk grouping system for the postoperative prognosis of IMPC patients, further demonstrating the application of this prognostic model.

## 4 DISCUSSION

BC is the most common form of cancer and the leading cause of cancer deaths in women worldwide.^11^ The combination of various treatment modalities, such as chemotherapy, hormone therapy, targeted therapy and immunotherapy, can effectively control disease progression and improve patients' quality of life.^12^ Many patients with IMPC have associated clinically disease‐positive lymph nodes, often related to the strong lymphovascular tropism of tumor.^13^ The potential biology of microcapillary histological patterns is detrimental in the lymphatic directionality of tumors.^13^ Verras et al. suggested that although there are no specific guidelines, breast surgeons should be aware that IMPC may require more extensive marginal excision.^13^

Although IMPC has a high propensity for lymph node metastasis, various studies have shown that its overall prognosis is similar to that of IDC.^9^, ^14^, ^15^, ^16^ Comparison of IMPC and IDC using propensity score matching (PSM) to remove confounding factors revealed no significant differences in OS and disease‐free survival (DFS) between the two groups.^16^ The BCSS and OS of IMPC were even superior to those of IDC in AJCC stage II‐III, and histology grade II‐III.^17^ Chen et al. proposed that the disease‐specific survival (DSS) and overall prognosis of IMPC are similar to those of IDC and that patients with ER‐negative or ER‐positive lymph nodes ≥4 have the worst prognosis.^14^ In addition to positive ER and fewer lymph nodes, Lewis et al. further demonstrated that age <65 years and receipt of radiotherapy were also protective factors.^4^ Ye et al. analyzed 1407 IMPC patients from the SEER database and found that larger tumors, younger age, black race, and lack of hormone receptor expression were significantly associated with regional lymph node involvement.^18^ Surgery can effectively alleviate the progression of IMPC. However, there is no prognostic prediction for patients with IMPC after surgery. Nomograms are conducive to the promotion of personalized medicine and have been proposed as a means to improve disease prediction.^19^, ^20^, ^21^

In this study, we analyzed 1855 IMPC breast cancer patients from the SEER database after surgery and identified age, race, marital status, stage, ER status, radiotherapy and chemotherapy as factors affecting prognosis. The above characteristics were further used to build a nomogram for predicting the 3‐, 5‐, and 10‐year OS. Unfavorable prognostic factors for IMPC patients after surgery included age ≥62, black race, stage III‐IV and negative ER status. Receiving radiotherapy or chemotherapy improved patient prognosis, while there was no difference in OS between BCS and mastectomy. The AUC values of the training and validation sets, as well as the C‐index, demonstrated the strong accuracy of the model in predicting 3‐, 5‐, and 10‐year OS. The calibration curves proved the consistency of the model's prediction results with the actual situation, while the results of DCA further suggested that this model has good clinical applicability. In general, our model has good prediction performance in predicting the postoperative prognosis of IMPC patients, which can provide guidance to clinicians in determining the severity of disease and formulating treatment plans.

This study is a retrospective study and inevitably has some limitations. Information on the HER2 status of tumors prior to 2010 was not available for 31.2% of the total population, thus potentially ignoring the prognostic impact of HER2 status. Although M stage was statistically significant in the univariate analysis, we did not include M stage in the multivariate analysis because of the bias of the results due to the small sample size for the occurrence of distant metastases. Overall, our model has good clinical applicability in predicting the postoperative prognosis of IMPC patients.

## 5 CONCLUSIONS

In this study, we used the SEER database for the first time to analyze prognostic data in patients with IMPC in breast cancer after surgery. A nomogram of estimated OS at 3, 5, and 10 years was created based on a large study cohort. The current model has good predictive power for patient diagnosis, risk assessment and clinical decision‐making, thus helping clinicians provide highly customized patient management in the future.

## AUTHOR CONTRIBUTIONS

**Yuyuan Chen:** Conceptualization (equal); data curation (equal); formal analysis (equal); funding acquisition (equal); investigation (equal); methodology (equal); project administration (equal); resources (equal); software (equal); supervision (equal); validation (equal); visualization (equal); writing – original draft (equal); writing – review and editing (equal). **Caixian Yu:** Conceptualization (equal); data curation (equal); formal analysis (equal); funding acquisition (equal); investigation (equal); methodology (equal); project administration (equal); resources (equal); software (equal); supervision (equal); validation (equal); visualization (equal); writing – original draft (equal); writing – review and editing (equal). **Dedian Chen:** Conceptualization (equal); data curation (equal); formal analysis (equal); funding acquisition (equal); investigation (equal); methodology (equal); project administration (equal); resources (equal); software (equal); supervision (equal); validation (equal); visualization (equal); writing – original draft (equal); writing – review and editing (equal). **Yiyin Tang:** Conceptualization (equal); data curation (equal); formal analysis (equal); investigation (equal); methodology (equal); project administration (equal). **Keying Zhu:** Software (equal); supervision (equal); validation (equal); visualization (equal); writing – original draft (equal); writing – review and editing (equal). **Rong Guo:** Resources (equal); software (equal); supervision (equal); validation (equal); visualization (equal); writing – original draft (equal); writing – review and editing (equal). **Sheng Huang:** Conceptualization (lead); data curation (equal); formal analysis (equal); funding acquisition (equal); investigation (equal); methodology (equal); project administration (equal); resources (equal); software (equal); supervision (equal); validation (equal); visualization (equal); writing – original draft (equal); writing – review and editing (equal). **Zheng Li:** Conceptualization (equal); data curation (equal); formal analysis (equal); funding acquisition (equal); investigation (equal); methodology (equal); project administration (equal); resources (equal); software (equal); supervision (equal); validation (equal); visualization (equal); writing – original draft (equal); writing – review and editing (equal). **Lvjun Cen:** Conceptualization (equal); writing – review and editing (equal).

## FUNDING INFORMATION

This work was supported by the National Natural Science Foundation of China (81860465 and 8216110588).

## CONFLICT OF INTEREST

The authors declare that there are no potential conflicts of interest disclosed.

## RESEARCH INVOLVING HUMAN PARTICIPANTS AND/OR ANIMALS

This article does not contain any studies with human participants or animals performed by any of the authors.

## Data Availability

This study used de‐identified data from the National Cancer Institute's Surveillance, Epidemiology and End Results program without consent. All data generated or analyzed during this study are included in this published article.
